# Anaerobic sequencing batch reactor enhances short-chain fatty acids productivity while facilitating downstream processing

**DOI:** 10.3389/fmicb.2026.1873229

**Published:** 2026-08-19

**Authors:** Marta de Vicente, Elvira Romero, Elia Tomás-Pejó, Cristina Gonzalez-Fernández

**Affiliations:** 1 Biotechnological Processes Unit, IMDEA Energy, Móstoles, Spain; 2 Faculty of Biological Sciences, Universidad Complutense de Madrid, Madrid, Spain; 3 Department of Chemical Engineering and Environmental Technology, School of Industrial Engineering, University of Valladolid, Valladolid, Spain; 4 Institute of Sustainable Processes, Valladolid, Spain

**Keywords:** acetate kinase, anaerobic fermentation, butyrate kinase, food waste, microbial population analysis, sequencing batch reactor, short-chain fatty acids

## Abstract

Anaerobic fermentation (AF) emerged as a promising technology to produce short-chain fatty acids (SCFAs). However, downstream processing of SCFAs is often challenging and costly due to the complexity of AF effluents that present high solids content. Sequencing batch reactors (SBRs) promote effluent separation in two phases (solids and supernatant), which can be an advantage to facilitate SCFAs downstream processing. This work evaluated the performance of SBRs operational configuration at different hydraulic retention times (HRT) (20, 15 and 10 d). The maximum SCFAs concentration of 28.3 g/L was reached at HRT of 20 d, whereas the maximum bioconversion (60.7%) and productivity (1.8 g SCFAs /Ld) were reached at HRT of 10 d. Regardless of the HRT, the predominant SCFAs found at the steady state were butyric and acetic acids (> 70% w/w), in accordance with the detection of acetate and butyrate kinases in all cell lysates. The SBR configuration enabled a 1.3-fold increase in the SCFAs yield (g SCFAs/g TS_effluent_) when comparing the supernatant and the solids phase. The effect of different HRT was analyzed to establish the correlation between SCFAs production yield and the different microbial populations developed in the reactors. Despite the different HRT, *Clostridium*, *Atopobium*, and *Bulleidia* were the predominant genera in all cases, demonstrating their key role in SCFAs production. These results shed light on the HRT effect to promote high SCFAs accumulation and bioconversion efficiencies under SBR configuration, while easing SCFAs recovery and improving waste revalorization.

## 1 Introduction

The growing global population has led to a marked increase in food demand, and consequently, to an important generation of food waste (FW). According to the United Nations Environment Programme (2024), 1.05 billion tons of FW are generated worldwide yearly. Identifying cost-effective and sustainable strategies for the treatment and valorization of FW is crucial to minimize the environmental impact of waste disposal while contributing to the generation of high value-added biochemicals. Anaerobic fermentation (AF), has emerged as a promising technology to convert complex organic matter into short-chain fatty acids (SCFAs) (Gonçalves et al., 2024). Carboxylates range from 2 to 6 carbons. While C2–C5 compounds are generally classified as SCFAs, C6 has also been categorized as a medium-chain FAs (MCFAs) (Battista et al., 2024; Spirito et al., 2014). For consistency, all acids have been classified as SCFAs in the present study. SCFAs have several industrial applications, including the production of biofuels, bioplastics and biosurfactants (Sun et al., 2024; Tomás-Pejó et al., 2023).

FW composition, process pH, temperature, organic loading rate (OLR), hydraulic retention times (HRT) are some of the most important parameters to consider when targeting at SCFAs production via AF (Aboudi et al., 2023; Greses et al., 2022a; Afecto Gonçalves et al., 2025). With regard to reactor configurations, continuous stirred-tank reactors (CSTR) have been often the preferred choice for organic matter revalorization given their low operational complexity. However, the presence of solids hampers downstream processing when the target product is soluble (i.e., SCFAs) and needs to be extracted from the effluent for further usage. Lowering the solids content in SCFAs-rich effluents would allow a cheaper and less complex downstream process, which actually accounts for up to 50% of the total process cost (Chen et al., 2022).

Sequencing batch reactors (SBR) are operated in 4 sequential steps; namely feeding, reaction, settling and decanting. This mode of operation allows the separation of the supernatant from the solid phase during the settling step. SBRs, that have been mainly used as alternatives to the conventional activated sludge for nutrients removal from wastewater (Blackburne et al., 2008; Qi et al., 2019), are particularly useful when treating high-strength industrial wastewater. However, only a limited number of studies have focused on the AF of organic wastes in SBR for SCFAs production and those available have primarily been conducted using synthetic substrates or non-agricultural feedstocks (Karthikeyan et al., 2016; Santiago et al., 2019; Simonetti et al., 2023).

This study evaluated the use of SBR to increase SCFAs productivity and bioconversion efficiency, while simultaneously reducing the solid content in the effluent to facilitate downstream processing. To this end, different HRT (10, 15 and 20 d) were assessed to identify the optimal conditions to revalorize carbohydrate-rich FW into SCFAs. Microbial analysis in terms of diversity, abundance and distribution were also carried out to stablish the relationship between the microbial community developed in the SBRs and process performance in terms of product yields.

## 2 Materials and methods

### 2.1 Feedstock and inoculum

The FW composed by a mixture of vegetables and fruits containing cucumber, tomato, red pepper, zucchini and melon was provided by an agricultural company (Cajamar) in Sta. Maria del Águila-El Ejido, Almería (Spain). Each vegetable and fruit contributed to the mixture in equal quantities (23.8% w/w each), except for the red pepper that only accounted for 5% w/w. The mixture was blended, homogenized, and stored at −21 °C to avoid self-fermentation. The feedstock was characterized in terms of pH, total and volatile solids (TS and VS, respectively), total and soluble chemical oxygen demand (TCOD and SCOD, respectively), ammonium (NH_4_^+^-N), acetic acid, citric acid, lactic acid, carbohydrates, lipids, proteins, and ash (Table 1). The mean and standard deviation of the feedstock composition were calculated from measurements taken each time a new batch was prepared, considering all experimental days (*n* = 62).

**TABLE 1:** FW characterization.

	Agricultural FW	
	Mean	SD
pH	4.6	0.2
TCOD (g/L)	84.7	5.4
SCOD/TCOD (%)	76.4	5.6
TS (g/L)	66.3	0.7
VS/TS (%)	91.5	0.5
NH_4_^+^-N (g/L)	0.1	0.01
Ash (%)	8.5	0.1
Lipids (%)	5.7	1.0
Proteins (%)	12.9	2.4
Carbohydrates (%)	70.3	2.5
Acetic acid (g/L)	1.0	0.2
Citric Acid (g/L)	11.5	< 0.1
Lactic Acid (g/L)	4.2	< 0.1

Anaerobic sludge collected from a wastewater treatment plant (WWTP) in Móstoles, Madrid (Arroyo de El Soto) was used as inoculum in all cases. The inoculum had the following composition: 19.0 ± 0.6 g/L TS and 21.1 ± 1.0 g/L TCOD, with a VS/TS ratio of 67 ± 0.5% and a SCOD/TCOD ratio of 4.1 ± 0.6%. NH_4_^+^-N concentration of 0.71 ± 0.01 g/L and a pH of 7.7 ± 0.1. The mean and standard deviation of the anaerobic sludge composition was calculated by analyzing the initial composition in triplicate.

### 2.2 Anaerobic reactors configuration. Experimental set up

Experiments were carried out in three SBRs with 3 L of working volume. AF conditions were stablished at 25 °C (controlled with a water bath, Julabo CD-0200F) and pH ranging between 5.8 and 6.0 by manually adding NaOH (5 M). These conditions were selected to enhance the acidogenic and acetogenic steps of the AF and to reduce methanogenic activity, according to previous results (Greses et al., 2020; Liu et al., 2023). To guarantee anaerobic conditions, the headspace of all SBRs was purged with helium for 30 min. The stirring was set continuously at 80 rpm using a Heidolph stirring (Hei- TORQUE Expert 200). The three reactors were operated with an OLR of 3 g VS/Ld. These conditions were selected based on Gonçalves et al. (2024) for optimum bioconversion of complex FW. Reactors were run at HRT 20, 15 and 10 d (named HRT20, HRT15 and HRT10, respectively). The feedstock was diluted with tap water to reach the desired VS, according to the OLR and the HRT imposed for each reactor.

All reactors were allowed to settle overnight (approx. 12 h). For HRT20, the media was mixed to ensure complete homogenization before medium withdrawal and feeding. After the feeding, HRT20 was stirred until the settling time. For HRT20, the longer HRT minimized the effect of solid-liquid separation. HRT15 and HRT10 were not homogenized before medium withdrawal to ensure two-phase separation (Table 2 and Supplementary Figure 1). This allowed the removal of different volumes from the solid and supernatant phase depending on the process conditions (different evaluated HRT). Therefore, HRT15 and HRT10 were the applied experimental conditions demonstrating the solid-liquid separation advantages of SBR. A total of 150 mL was withdrawn daily from HRT20. For HRT15, 150 mL was collected from the sludge and 50 from the supernatant, whereas 150 mL from each phase was collected for HRT10 (Supplementary Figure 1). All SBRs were run for 62 d, until steady state was achieved and constant values of COD, NH_4_^+^-N, SCFAs, etc. were determined in the effluents. The steady state was reached after approximately 40 d.

**TABLE 2:** Operational phases and time periods in the SBR configuration.

	Feed (min)	Reaction (min)	Settling (min)	Mix (min)	Sample (min)
HRT20	10	720	680	20	10
HRT15/HRT10			700	–	

### 2.3 Analytical methods

Sample analysis was done separately to each phase collected from the SBRs (solids and supernatant phases). Samples were analyzed twice per week to control AF performance. pH was daily measured using a pH-meter GLP21 (Crison, Hach Lange). TS, VS and ash were determined following the Standard Methods procedures (APHA, 2017). TCOD, SCOD and NH_4_^+^-N were determined using commercial kits ISO 15705 and ISO 000683 (Merck), respectively. Carbohydrates were determined according to the phenol-sulfuric method (Dubois et al., 1956). Total Kjeldahl nitrogen (TKN) was analyzed using the 4500-N_*org*_ Standard methods procedure (APHA, 2017), and protein content was calculated by multiplying the nitrogen value by 6.25 (López et al., 2010). Lipids percentage was determined gravimetrically by using the Folch method (Folch et al., 1957) with some modifications. 200 mg of dry FW biomass were resuspended in 10 mL HCl (5 M) and then incubated at 78 °C for 2 h in a water bath. After cooling, 5 mL of chloroform and 10 mL of methanol were added. Afterward, 5 mL of chloroform and 5 mL of 9 g/L of NaCl solution were added and stirred again for 10 min at room temperature. The mixture was centrifuged for 10 min at 3,500 × *g*. The chloroform phase was then transferred into a weighted flat bottom Erlenmeyer and evaporated with nitrogen. Lastly, Erlenmeyer’s were weighted to calculate the total lipid content.

SCFAs were analyzed by liquid chromatography using an Agilent 1260 HPLC_RID equipped with Cation H Refill Catridge Microguard column (Biorad, Hercules, CA, USA) and an Aminex HPX-87H ion exclusion column (300 * 7.8 mm I.D., Biorad). The mobile phase was 5 mM H_2_SO_4_ solution. Elution was conducted at a flow rate of 0.6 mL/min. 0.5 mL of samples were filtered using a 0.22 μm nylon filter (Branchia). The injected sample volume was 20 μL. The oven and detector temperatures were 25 and 35 °C, respectively. Gas production was also daily measured using a flow meter (Bioprocess Control, Sweden). A gas chromatograph with a thermal conductivity detector (Clarus 580 GC, PerkinElmer) and two columns (HSN6-60/80 Sulfinert P 7’ “x 1/8”’ O.D. and MS13 * 4-09SF2 40/60P 9“x 1/8”’ O.D., PerkinElmer) was used for gas composition measurement.

### 2.4 Calculations

Process efficiency was assessed by measuring: (i) the organic matter bioconversion into SCFAs (Equation 1), (ii) the acidification efficiency of SCOD (Equation 2), (iii) the COD removal, which refers to the conversion of the feedstock TCOD into biogas (Equation 3), and (iv) the hydrolysis efficiency based on the percentage of VS removal (Equation 4). These parameters allowed evaluating process evolution and efficiency, together with the process productivity (Equation 5). COD and VS balances were done separately for both phases (solid and supernatant). Mineralization was also calculated to evaluate the conversion of proteins (organic nitrogen) into NH_4_^+^-N (Equation 6). The mean and standard deviation were calculated based on the samples collected at multiple consecutive time points during the steady state (*n* = 7).

In Equations 1, 2, SCFAs effluent is the sum of all SCFAs (acetic, propionic, butyric, iso-butyric, valeric, iso-valeric and caproic acids) determined in the samples in terms of COD. These acids were converted into their COD equivalents by applying their respective conversion factors, which corresponds to 1.066, 1.512, 1.816, 1.816, 2.037, 2.037 and 2.2, respectively.


$$\%Bioconversion=\frac{\sum C\,O\,D-S\,C\,F\,A\,s_{\text{effluent}}}{{\mathrm{TCOD}}_{\mathrm{influent}}}•100$$ (1)

%⁢A⁢c⁢i⁢d⁢i⁢f⁢i⁢c⁢a⁢t⁢i⁢o⁢n= (2)

 ⁢∑C⁢O⁢D-S⁢C⁢F⁢A⁢seffluent-SCFAsinfluentSCODeffluent•100

%COD(TCODinfluent-TCODeffluent)TCODinfluentr⁢e⁢m⁢o⁢v⁢a⁢l=•100 (3)

%VS=r⁢e⁢m⁢o⁢v⁢a⁢lVSinfluent-⁢VSeffluentVSinfluent•100 (4)

$$P\,r\,o\,d\,u\,c\,t\,i\,v\,i\,t\,y\,\left(g/L\,d\right)=\frac{\mathrm{SCFAs}\,\left(g/L\right)}{\mathrm{HRT}\,\left(d\right)}$$ (5)

$$Mineralization\left(\%\right)=\frac{\left(\left({\mathrm{NH}}_{4}^{+}-N\,\mathrm{effluent}-{\mathrm{NH}}_{4}^{+}-\text{Nin}\right)\right)}{\left(\left(\text{TKNin}\right)-\left({\mathrm{NH}}_{4}^{+}-\mathrm{Nin}\right)\right)}$$ (6)

To assess the statistical significance of the results, a one-way ANOVA analysis was carried out with a 95% confidence interval, considering significant difference at a *p*-value < 0.05. Data collected from each reactor during steady-state (*n* = 7) were used for the statistical analysis. Leuven’s and Shapiro–Wilk test were carried out to confirm the normality and homoscedasticity of the samples (*p*-value > 0.05). Pairwise comparison was conducted via Tukey test. All analysis were carried out using R software (4.3.2).

### 2.5 Microbiological community analysis

16S RNA genes were analyzed to evaluate the differences between the inoculum and the microbial communities developed upon the different HRT implemented in the SBRs. 1 mL of the final sample (62 d) was used for DNA extraction using FastDNA SPIN kit for Soil (MP Biomedicals, LCC). DNA quality and quantity were measured with a nanodrop plate reader (SPECTROstar Omega, BMG LABTECH). V3 and V4 regions of the 16S rRNA for both bacteria and archaea were targeted using the 341F and 805R (F—CCT ACGGG NGGC WGC AG and R—GAC TAC HVGGGTAT CTA ATC C) primers. Sequencing was done by FISABIO (Valencia, Spain) in a Miseq sequencer by merging the paired-ends from each sample through the PEAR program and followed by sequence filtering those with a mean quality greater than 30 with PRINSEQ. The primer sequences were eliminated with Mothur (Schloss et al., 2009). The chimeric sequences were also removed with the Quantitative Insights Into Microbial Ecology (QIIME) 1.9.1 software package and the final sequences at 97% were grouped into operational taxonomic units (OTUs). Taxonomic annotation was assigned using the Greengenes database (v13.8). In order to evaluate the evenness and richness of the samples, alpha diversity analysis following the Shannon index was calculated using QIIME. Alpha and beta diversity indexes helped evaluating the species variability within a sample and the composition differences between samples, respectively. Data were statistically analyzed using canonical correspondence analysis (CCA) to stablish the correlation between the SCFAs concentration and the developed microbial community in the reactors using PAST4 (Hammer et al., 2001).

### 2.6 Enzymatic activities

Acetate kinase (AK, EC 2.7.2.1) and butyrate kinase (BK, EC 2.7.2.7) activities were measured for the sludge (0 d) and at the beginning (12 d), middle (32 d) and end (62 d) of the AF in the reactors operated at the three different HRTs. At each sampling point, three separate 2 mL samples were collected and processed independently in duplicates. Each sample was centrifuged (14,700 rpm, 5 min, room temperature) to remove the supernatant. 0.1 g of pellet was resuspended in 196.6 μL of 0.1 M Tris-HCl and then mixed with 24.4 μL of the lysis buffer MT, before being transfered to the Lysing Matrix E tube (FastDNA^®^ Spin Kit for Soil, MP Biomedicals), and subjected to bead-beating using a FastPrep^®^ instrument (6 cycles, each 30 s at 6 M/S and 5 min in ice). After the lysis and subsequent centrifugation (14,700 rpm, 7.5 min, room temperature), the supernatant was mixed with 800 mM acetate or butyrate, 10 mM ATP, 10 mM MgCl_2_, 50 mM Tris-HCl pH 7.0, and 700 mM hydroxylamine hydrochloride (25 μL each component to reach the indicated final concentrations). The equilibrium of these reactions was driven toward the acetylated product (forward direction) by the hydroxylamine (prepared at pH 7.0 with NaOH), given its reaction with the acetylated product forming hydroxamate. After the incubation at 60 °C for 6 min, reactions were stopped and an orange-brown complex between the hydroxamate product and ferric iron was formed by adding 50 μL of a mixture of trichloroacetic acid and FeCl_3_-HCl (1:1) (final concentrations of 0.459, 0.063, and 1.250 M, respectively) (Fowler et al., 2011; Liu and Steinbüchel, 2000; Rose, 1955). The absorbance at 540 nm of these samples was determined in a plate reader (SPECTROstar Omega, BMG LABTECH), corrected by subtracting the control value without the cell lysate, and used to determine the μmols of product based on the Beer-Lambert law and a previously determined extinction coefficient of 828 and 169 M^–1^cm^–1^ for the AK and BK, respectively (Liu and Steinbüchel, 2000; Wofford et al., 1986). One enzymatic unit (1 U) for the kinase assays is defined as the amount of enzyme that catalyzes the formation of 1.0 μmol of acetyl or butyryl phosphate per mL under the specific assay conditions. In the same samples, total protein concentration of the cell lysates was determined using the BCA Protein Assay Kit (Novagen^®^) and bovine serum albumin as a standard to calculate the kinase U per mg of total protein in the lysate. A two-way ANOVA was carried out to assess the differences in enzymatic activity between days and HRT conditions after verifying normality (Shaphiro–Wilk test) and homoscedasticity (Levene’s test). Pairwise comparison was conducted via Tukey test, using R software (4.3.2). As the inoculum is the same for all three reactors it was not included in the analysis.

## 3 Results and discussion

### 3.1 HRT effect on process performance when targeting at SCFAs production

Hydrolysis is responsible for breaking down the organic matter into simpler compounds (sugars, amino acids and long-chain fatty acids), which are subsequently converted into SCFAs and other metabolites during the acidogenesis step. More specifically, the VS removal is used as an indicator of the hydrolysis efficiency while the acidification shows the conversion of the soluble intermediates into SCFAs during the acidogenesis. Despite the general idea that long HRT (> 20 d) mediates a favorable hydrolysis (Bolaji and Dionisi, 2017), only slight differences where observed in the present study in terms of VS removal when comparing different HRT (20, 15, 10 d) (Table 3). In this study, SBRs operated at three different HRT allowed for 40–45% hydrolysis efficiencies. These values were similar to those achieved by Gonçalves et al. (2024) when employing similar operational conditions (pH 6, OLR 3 g VS/Ld, HRT = 20 d) in a CSTR. It can be thus inferred that the organic matter reached an apparent limit determined by the degradability of the substrate. Consequently, increasing the HRT did not further enhance the VS removal which was more influenced by the fixed OLR and the substrate composition. Also, the high SCOD/TCOD of the effluents showcased the organic fraction available for the subsequent acidogenic stage (> 60% for all reactors, Table 4), corroborating an efficient hydrolysis step. All tested HRTs promoted an almost complete transformation of SCOD into SCFAs with more than 94% acidification efficiency (Table 3), confirming that the operational conditions were particularly suitable for SCFAs production. The slightly acidic pH (5.8) favored acidogenesis instead of methanogenesis, reducing the SCFAs consumption by acetogenic microorganisms and promoting its accumulation in the system. The efficiency of the acidogenesis phase was also evidenced by the low COD removal (Table 3), indicating minimal methane production (approximately 25% of the total gas produced was determined to be methane, with the remaining 75% corresponding to carbon dioxide). COD mass balance was also calculated for each reactor, showing a closure of 85–97%, being consistent with the COD removal.

**TABLE 3:** Effluents characterization for each reactor.

	HRT 20		HRT 15		HRT 10	
	Mean	SD	Mean	SD	Mean	SD
VS_removal_ (%)*	44.7*^A^*	4.7	51.3*^B^*	2.4	40.4*^A^*	3.5
Acidification (%)	96.6	4.2	98.4	5.9	95.3	4.0
Total SCFAs (g /L)*	28.3*^C^*	0.7	23.4*^B^*	0.5	17.6*^A^*	0.4
Bioconversion (%)*	59.1*^B^*	1.8	54.8*^A^*	2.2	60.7*^B^*	2.2
NH_4_^+^-N (g N/L)*	0.4*^C^*	0.04	0.3*^B^*	0.02	0.1*^A^*	0.01
COD_removal_ (%)*	6.0*^A^*	1.9	15.5*^C^*	3.0	10.4*^B^*	4.3

The values were calculated through a mass balance between both phases. The asterisk (*) indicates a statistically significant overall effect of the HRT on each parameter (one-way ANOVA, *p* < 0.001). The letters indicate which condition significantly differ from the others according to Tukey-adjusted pairwise comparisons (*p* < 0.05). Values sharing the same letter are not significantly different.

**TABLE 4:** Composition of the SBR effluents during the steady state of the fermentation.

	HRT20	HRT15 solids	HRT15 supernatant	HRT10 solids	HRT10 supernatant
TCOD (g/L)	77.6 ± 2.6	64.2 ± 4.3	46.1 ± 0.8	51.0 ± 3.5	34.4 ± 2.1
SCOD/TCOD (%)	64.6 ± 3.3	62.1 ± 6.8	81.8 ± 3.9	58.8 ± 4.8	86.7 ± 4.5
TS (g/)	50.2 ± 1.1	42.9 ± 1.3	31.8 ± 0.9	36.4 ± 2.6	24.3 ± 0.5
VS (g/)	32.5 ± 1.3	28 ± 1.4	17.5 ± 0.9	22.6 ± 3.1	13.1 ± 0.4
SCFAs/TS_effluent_ (g/g)	0.6 ± 0.02	0.5 ± 0.02	0.7 ± 0.03	0.5 ± 0.04	0.7 ± 0.02

**FIGURE 1:**
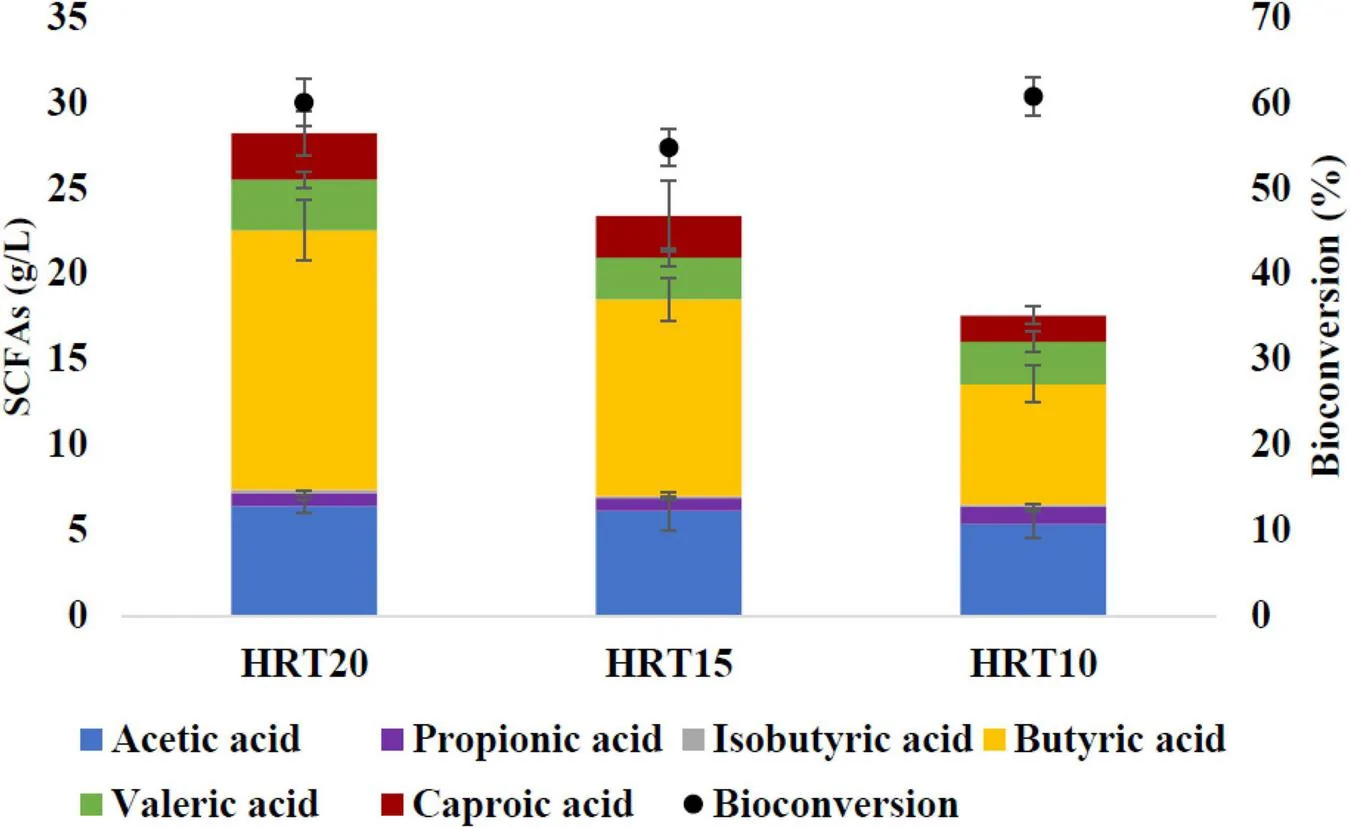
SCFAs profile (g/L) and bioconversion efficiencies (%) at the steady-state for all three reactors at an HRT of 20, 15 and 10 d. *p*-value = 1.37e^–17^.

HRT20 achieved the highest SCFAs production in terms of concentration (28.3 ± 0.7 g/L) when compared to HRT15 and HRT10 (23.4 ± 0.5 g/L and 17.6 ± 0.4 g/L, respectively) (Table 3 and Figure 1). These concentrations were equivalent to 48.2 ± 1.2, 39.2 ± 0.8 and 28.8 ± 1.2 g COD/L, respectively. Despite the different SCFAs concentrations, bioconversion efficiencies reached 59.1 ± 1.8, 54.8 ± 2.2 and 60.7 ± 2.2%, for HRT20, HRT15 and HRT10, respectively. These similar bioconversion efficiencies were the result of applying the same feedstock dilution to maintain the OLR and HRT. The attained bioconversions were higher than those typically reported for AF of organic wastes in CSTR reactors (i.e., 25–50%) (Greses et al., 2020, 2022b; Yu et al., 2021). These results were attributed to the agricultural waste degradability. Within the tested range, shorter HRT was not a limiting factor for hydrolysis not acidogenic conversion as seen in the comparable VS removal, acidification and bioconversion efficiencies. The microbial community even converted all the organic fraction more efficiently in less time indicating that extended HRT are not needed when treating agricultural waste. Conversely, a higher SCFAs concentration was obtained at HRT20. This was attributed to the higher VS_in_ concentration required to maintain the same OLR at longer HRT, rather than an improvement in bioconversion efficiency.

The bioconversion reached by HRT20 (59.1 ± 1.8%) was significantly higher than that achieved in previous reports in CSTR (49.2 ± 2% in CSTR) under the same HRT and similar FW composition (Gonçalves et al., 2024). Greses et al. (2023) obtained a similar bioconversion efficiency in CSTR (i.e., 59.6 ± 0.5%) with melon waste at HRT of 28.1 d, compared to the present study. The high efficiency reported in CSTR was obtained at the same pH and OLR as in this study, but with higher HRT (28 d), evidencing once again that SBRs enabled similar bioconversion efficiencies at lower HRT.

**FIGURE 2:**
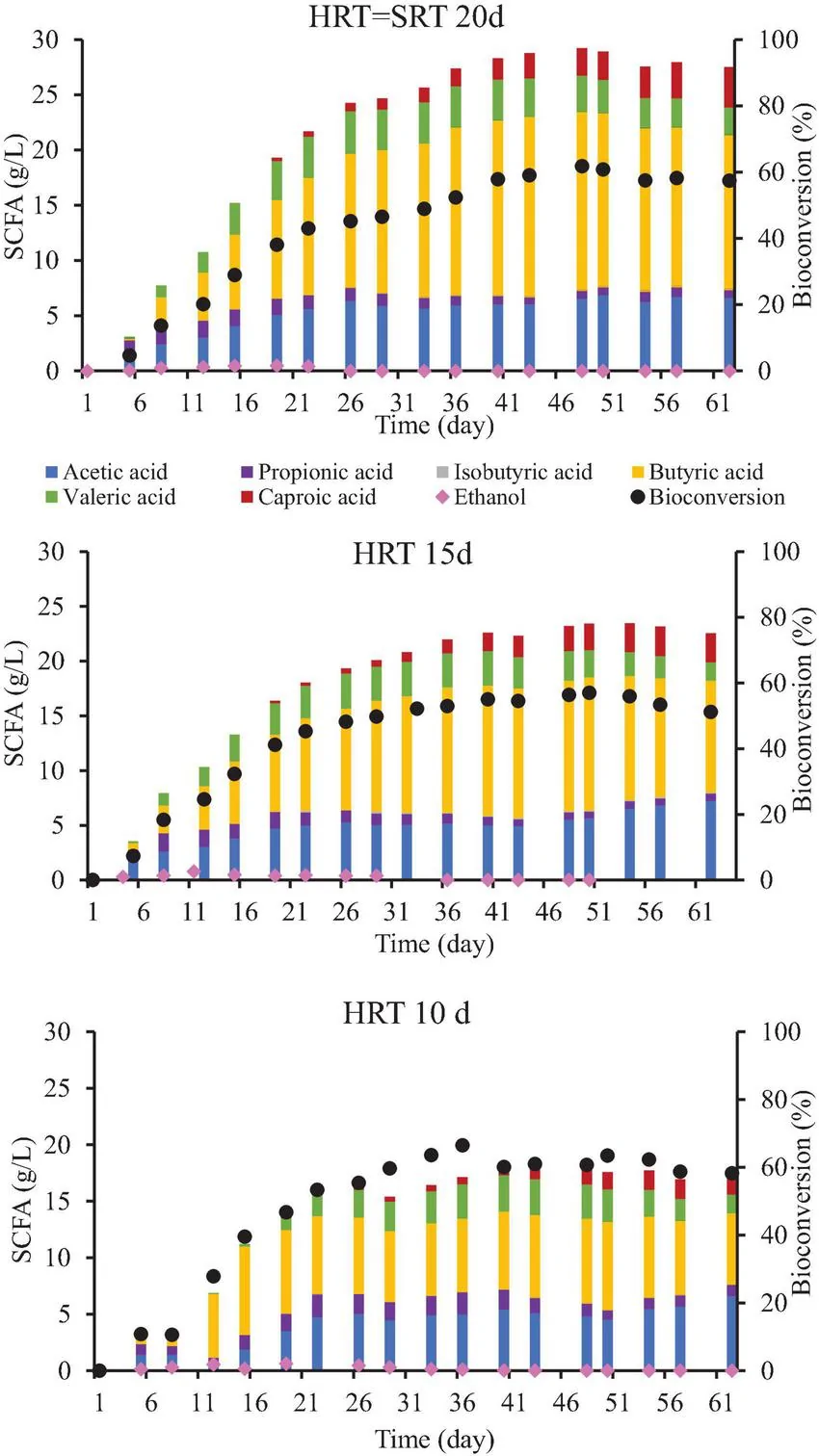
SCFAs concentration evolution during AF at three different HRTs.

Although different SCFAs concentration were reached (Table 3), the distribution profile was similar with a prevalence of butyric acid, followed by acetic, valeric and caproic acids (Figure 1). The SCFAs concentration evolution for the three AF reactors is presented in Figure 2. The selectivity toward acetic and butyric acids production (approx. 70% of the total SCFAs pool) could be attributed to the high carbohydrate content in the feedstock (70.3 ± 2.5% w/w, Table 1) and the slightly acidic pH (5.8–6) imposed in the SBR. These acids prevalence agreed with those reported by Gonçalves et al. (2024) and Greses et al. (2023) when working with agricultural FW in CSTR. Lago et al. (2023) also showed a prevalence of butyric and acetic acids production at HRT of 20 d when working with carbohydrate-rich feedstocks in SBR. In the present study, butyric acid proportion increased concomitantly with longer HRT, being 53.5 ± 2.2, 48.4 ± 3.3 and 39.8 ± 2.5%, in HRT20, HRT15 and HRT10, respectively. By contrast, acetic acid followed the opposite trend, being 22.6 ± 1.2, 26.0 ± 4.0 and 30.2 ± 4.2% in HRT20, HRT15 and HRT10 (Figure 1). The SCFAs distribution differences may derive from changes in the primary AF microbial metabolic pathways taking place at different HRT. In this sense, butyric acid-type fermentation has been reported to prevail at longer HRT (Esquivel-Elizondo et al., 2017; Martínez-Mendoza et al., 2023). Slower-growing chain-elongating bacteria (e.g., *Clostridium*) convert acetate and an electron donor (e.g., ethanol, lactic acid) into medium-chain fatty acids via the reverse β-oxidation pathway, while faster-growing species at short HRTs rapidly convert the electron donor into different metabolites (e.g., acetate and hydrogen from ethanol) (Angenent et al., 2016). Accordingly, the highest butyric acid proportion and relative abundance of *Clostridium* species were observed at HRT20. However, the enhancement in butyric acid for HRT20 may be also the result of having a higher concentration of VS_in_ to maintain the same OLR at the three HRT.

When aiming at an economically feasible AF process, not only SCFAs yield but productivity should be also carefully considered. Operating at short HRTs, allows to treat more organic matter in the same period of time, which is indeed one of the advantages of the SBR. Thus, operating at short HRT in SBR may allow an increase in the organic waste input. This could enable the treatment of larger substrate loads, by increasing the OLR, as long as the system handling capacity is not exceeded. In the present investigation, SCFAs productivity (g SCFAs/Ld) increased when reducing the HRT, being 1.4 ± 0.05, 1.6 ± 0.04 and 1.8 ± 0.04 g SCFAs/Ld for HRT20, HRT15 and HRT10, respectively. These results highlighted again the benefits of working at the lowest HRT. However, decreasing the HRT does not always implies higher productivities, as HRTs below 5 d have been proven to compromise process stability and productivity in CSTR reactors (Lim et al., 2008; Llamas et al., 2022). Castro-Fernandez et al. (2024) reported a SCFAs productivity of 1.4–1.5 g SCFAs/Ld when working at pH 6 and HRT of 10 d in CSTR. These were lower productivities than the ones attained in this study for HRT10 d. This fact could be attributed to the different reactor configuration, but also to the high protein content of their FW feedstock, which might compromise efficient hydrolysis and acidogenesis steps.

The selected operational conditions led to a nitrogen mineralization of 22.3 ± 2.2, 25.6 ± 1.5 and 13.6 ± 1.6% for HRT20, HRT15 and HRT10, respectively. These significant differences observed between reactors, suggested that both the HRT extend had a great influence on nitrogen mineralization, which was enhanced by the extended contact period between microorganisms and substrate in HRT20 and HRT15. Nitrogen mineralization was considered to be low, which was in fact not a problem to attain high SCFA production yields. Proteolytic and oxidative enzymatic activities are known to be enhanced at slightly higher temperatures, suggesting that the applied 25 °C of the SBRs may not have been optimal for mineralization. In line with this, AF at 35 °C and HRT of 15 d and 40 d achieved 39% and 43% of nitrogen mineralization when using protein-rich feedstocks (Magdalena et al., 2021 and Barreiro-Vescovo et al., 2018).

All these results supported the fact that the SBR configuration enables high process efficiency (high bioconversion, productivity and SCFAs yield), when treating high organic loads. Despite the HRT decrease the robust microbial community maintained its functional capacity even at lower HRTs without compromising process performance. Also indicating that microbial washout was not a limiting factor under the tested conditions.

### 3.2 Downstream benefits of SBR configuration

As previously mentioned, one of the main advantages of SBR configuration is the difference in TS between the solids and supernatant phases after the settling step. When both phases were compared in HRT15 and HRT10 (Table 4), a 35% increment in SCFA yield (g SCFAs/g TS_effluent_) was observed between the supernatant and the solids phase. Few studies have discussed the advantages conferred by the SBR on the SCFAs yields and TS content in the effluents from FW, making comparison to other works difficult. In CSTR run at similar operational conditions, 0.56 g SCFA/g TS_effluent_ were reported at HRT of 20 d (Gonçalves et al., 2024), which was comparable with the SCFA yield achieved in the SBR operated at HRT20 (0.6 g SCFAs/g TS_effluent_, Table 4). This was explained by the absence of separation of the supernatant and solids phase. However, the SBRs operated at HRT15 and HRT10 achieved a ratio of 0.72 g SCFAs/g TS_effluent_ in the supernatant, leading to a 1.3-fold increase in SCFAs yield when compared to the solids phase (Table 4). When using the SBR, the higher g SCFAs/g TS yield achieved in the supernatant with lower TS would facilitate the SCFAs extraction and purification operation units, directly impacting the downstream processing. This beneficial effect is a particular feature of particulate feedstocks. SBR with other carbohydrate-rich feedstocks, such as molasses, at an HRT 20 d did not show significant differences in g SCFAs/g TS_effluent_ between the supernatant and solids phases, being 0.53 g SCFAs/g TS vs. 0.56 g SCFAs/g TS, respectively (Lago et al., 2023). This could be attributed to the viscous nature of the molasse feedstock that did not completely allowed two-phase separation. Because of that, not only feedstock composition but physical nature should be carefully considered to fully exploit the benefits of SBR.

A preliminary economic analysis was conducted to evaluate the advantages of SBR operation considering product recovery, SCFAs production and operational energy costs. The performance of SBR was compared with CSTR at the same HRT (Gonçalves et al., 2024). Energy requirements were estimated at 0.7–1.5 kWh/m^3^ (0.023–0.05 kWh/kg TS) for conventional industrial centrifuges (Fasaei et al., 2018; Szepessy and Thorwid, 2018), which may increase up to 0.12–0.26 kWh/kg TS when treating sewage sludge with a multi-disk roller or screw press systems (Chang et al., 2023). Considering average industrial electricity prices in the EU of 0.20–0.23 €/kWh (EUROSTAT, 2024), this corresponds to a processing cost of 0.13–0.35 €/m^3^ of centrifuged digestates. Compared to HRT20, lowering the HRT can result in downstream cost savings of 0.42–1.27 €/m^3^, given that SBR operation enables a reduction of around 20% in TS decrease compared to CSTR. However, SCFAs concentration should also be considered when evaluating overall process performance, as higher titers could improve the economic feasibility of the process. Additionally, SBR could reduce agitation time decreasing mixing energy consumption by up to 50%, as agitation is stopped overnight, which represents further saving of 20–80 €/m^3^/year, while also contributing to lower the capital costs of the process.

### 3.3 Abundance and statistical analysis of the microbial populations

**FIGURE 3:**
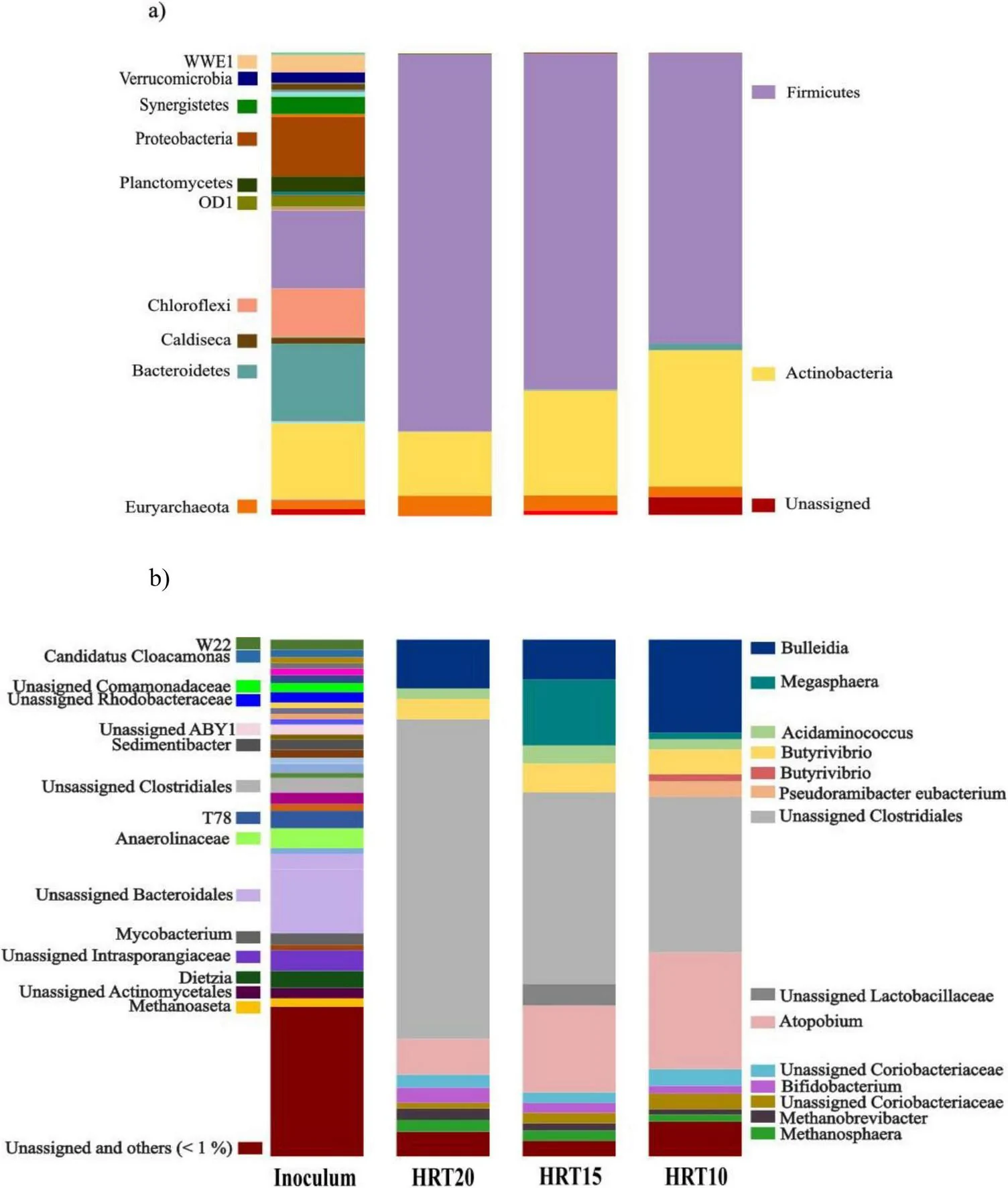
Relative abundance of the bacterial and archaea communities in the inoculum and SBR operated at different HRT at phylum **(a)** and genus **(b)** levels. Relative abundance lower than 1% is not included in the figure and appears as “others (< 1%).”

The microbiome analysis evidenced a diverse microbial community in the inoculum (Figure 3a). The most abundant phyla were Bacteroidota (20%), Firmicutes (18%), Actinobacteroidota (13%), Chloroflexi (12%) and Proteobacteria (10%) (Supplementary Table 1). This high biodiversity of the inoculum was dictated by the origin of the anaerobic sludge, which was collected from a wastewater treatment plant operated at high HRT and 35 °C. As observed in Figure 3, the selected operational conditions, together with the different HRT and reactor configuration, led to a significant microbial diversity decrease in SBRs. This is a common feature previously reported in AF when using anaerobic microbiomes collected from conventional digesters (Gonçalves et al., 2024).

The differences, not only in microbial richness but also in evenness, were evidenced by the Shannon index. The latter decreased from 7.734 in the inoculum to 3.084, 4.150 and 4.678, in HRT20, HRT15 and HRT10, respectively (Table 5). The richness decreases reflected microbial specialization and adaptation to the new operational conditions (25 °C, pH 6) in all three reactors. Contrary to what it would be expected, HRT20 allowed the highest richness and evenness decrease based on the alpha diversity analysis, according to the observed operational taxonomic units (OTUs), and Shannon index (Table 5). Although long HRT and SRT are expected to promote a higher microbial diversity, enabling the coexistence of both slow- and fast-growing microorganisms, the richness decrease in HRT20 could be attributed to the higher concentration of VS_in_ required to maintain the same OLR. These conditions likely imposed stronger selective pressure and increased stress on the microbial community, leading to a reduced diversity. This was evidenced by Spearmen and Pearson analysis, showing a strong negative correlation between VS_in_ and both OTUs and Shannon index (ρ = −1 and *r* = −0.98, for each analysis, respectively). Although the diversity of microorganisms under the selected HRT conditions diminished when compared to the inoculum, the present microbial community exhibited a wide metabolic versatility. The microbiome was capable of carrying out a broad range of functional activities, including key steps like hydrolysis and acidogenesis (Table 3). The greater diversity observed in HRT10 could be attributed to the faster turnover of the soluble organic fraction, promoting higher competition among microorganisms for the substrate when compared to HRT20 and HRT15. This was also evidenced by other studies at longer HRT (27 d), where a decrease in microbial diversity was similarly observed under comparable operational conditions (Greses et al., 2021). These findings indicate that HRT20 tended to reduce microbial diversity in the reactors while promoting metabolic versatility that resulted in a stable SCFAs distribution.

**TABLE 5:** Biodiversity indexes calculated in both inoculum and SBRs samples.

	Inoculum	HRT20	HRT15	HRT10
OTUs	2,854	588	1,170	1,483
Shannon index	7.734	3.084	4.150	4.678

As a result of inoculating with sludge from conventional AD, archaea accounted for 2% of the total microorganisms (Figure 3a) and were responsible for the slight methane production registered in the reactors (35.9, 21.2 and 71.2 mL CH_4_/g VS_in_ for HRT20, HRT15 and HRT10, respectively). Besides archaea, Firmicutes and Actinobacteria that can produce hydrolytic enzymes for carbohydrates and protein degradation, respectively, being therefore linked with NH_4_^+^-N, SCFAs, CO_2_ and H_2_ production (Greses et al., 2021; Lv et al., 2021; Zhang et al., 2024), were the only phyla found in HRT20 and HRT15. The efficiency of these phyla could be corroborated with the high hydrolysis (36–45% VS removal) and bioconversion (54–61%) efficiencies determined in HRT20 and HRT15 (Table 3). These results agreed with those obtained by Greses et al. (2020) and Gonçalves et al. (2024), where Firmicutes and Actinobacteria were also the predominant phylum when an anaerobic microbiome collected from conventional AD was subjected to selective conditions (slightly acidic pH and lower temperatures) to perform AF.

Despite the presence of Proteobacteria and Chloroflexi in the inoculum and being among the predominant phyla involved in organic matter degradation, especially during AD, both were completely washed out in all SBRs. This could be attributed to the slightly acidic pH imposed in the SBR (pH 6), as both phyla are more prone to thrive in fermentations ranging from pH 7 to 10 (Atasoy et al., 2019; Greses et al., 2017; Liu et al., 2014). Bacteroidetes were also not found in HRT20 and HRT15, with just a 2% abundance at HRT10, similarly due to their pH preference (Haindl et al., 2021).

Firmicutes accounted for 88% of the total bacterial abundance in HRT20, out of which Clostridia was the predominant class (83%), and followed by *Bacilli* (13%) and Negativicutes (3%). Eubacteriaceae and Lachnospiraceae families were the most predominant for all SBRs within Clostridia. Within Lachnospiraceae, *Butyrivibrio* is a cellulose-degrader bacterium involved in butyric accumulation (Atasoy et al., 2018; Liang et al., 2021). The higher relative abundance of *Clostridium*, particularly members of Lachnospiraceae, at HRT20, supported the previous observation that butyric acid production via the acetyl-CoA pathway increases at prolonged HRTs (Vital et al., 2014). *Bacilli* (mainly the Erysipelotrichaceae family) are responsible for the decomposition of fat and carbohydrates for SCFAs production (Zhang et al., 2024). *Pseudoramibacter* (Figure 3b) presented at a maximum abundance of 3% and it has been previously correlated with butyric and caproic acids production from acetic and lactic acids (De Groof et al., 2021; Zhu et al., 2022).

*Bulleidia*, mainly associated with acetic, propionic and caproic acid production (Gulhane et al., 2017), presented a higher relative abundance in HRT10, which correlated with the acetic acid increase in this case, at the expense of a decrease in butyric acid. *Acidaminococcus* genera, usually involved in monosaccharides degradation for propionic and butyric acid production (Joyce et al., 2018), presented low abundance in all three SBR, with a slightly higher value for HRT15 (Figure 3b). HRT15 also promoted *Megasphaera* abundance, a chain-elongating bacterium involved in the production of butyric acid from lactic and acetic acids (Lagoa-Costa et al., 2020; Monteiro et al., 2022). *Megasphaera* increased at the expense of *Clostridium* and *Bulleidia* in HRT15, however, no significant changes in process efficiency nor differences in the SCFAs profile were seen. The similar overall profile observed in HRT15 compared to HRT20, despite the HRT influence on acetic and butyric acid distribution, was probably due to the ability of many microorganisms to perform similar metabolic pathways in AF (Feng et al., 2018; Greses et al., 2022a; Valdez-Vazquez and Poggi-Varaldo, 2009). Some authors consider that butyric and valeric acids production by *Megasphaera*, involved in chain elongation, are related to the consumption of reducing equivalents (NADH, H^+^, e^–^) produced during acetic acid production (e.g., glycolysis), which could explain the constant presence of these acids despite the decrease of *Bulleidia* and *Clostridium* (Soto-Cruz et al., 2002).

The Actinobacteria’s highest representative was the Coriobacteria class (Figure 3), where *Atopobium* is involved in carbohydrates degradation for lactic and acetic acid production (Lim et al., 2019). The increase of *Atopobium* from 5% in HRT20 to 15% in HRT10 (Figure 3) supported the higher presence of acetic acid in the profile distribution (30.5% w/w, Figure 1). This aligned with previous reports that showed an *Atopobium* abundance increase at short HRT (Greses et al., 2021).

**FIGURE 4:**
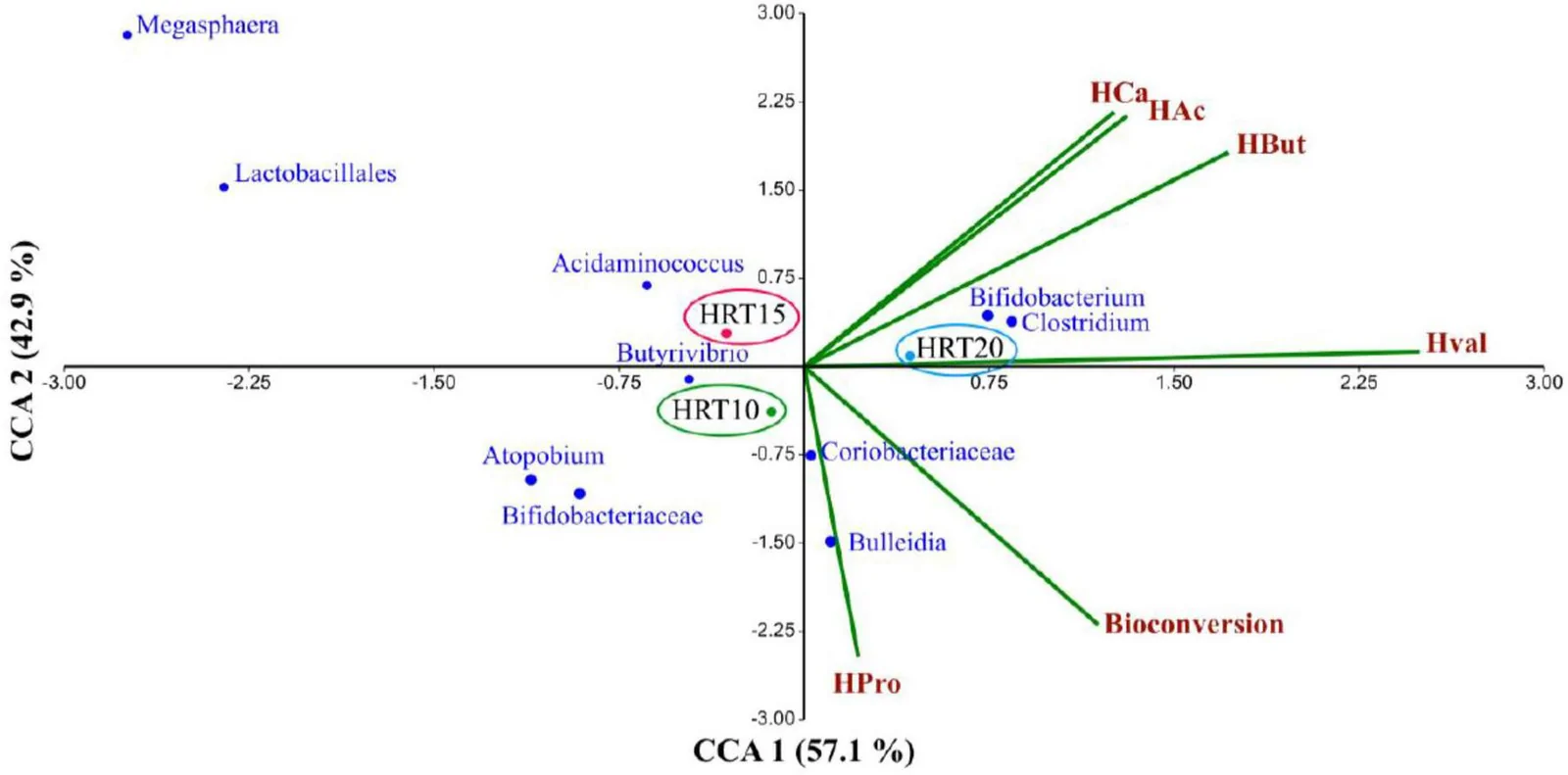
Canonical correspondence analysis (CCA) showing the relationship between different SCFAs production and microbial genus for the three different reactors. SCFAs and other reactor factors like bioconversion are displayed in green arrows. The different microorganisms are shown as blue dots. Only microorganisms with an abundance higher than 3% were considered for the CCA.

To explore potential association between operational conditions, the microbial community and the SCFAs production, a CCA at genus level was carried out. Each axis explained a percentage of the variables; namely, axis 1 explained 57.1% whereas axis 2 explained 42.9% of the total inertia. Both principal axis account for 99% of the variation; meaning that the plot remarkably summarized the relationship between samples. The triplot disclosed, at an exploratory level, the effect of the tested operational conditions on the developed microbial community (Figure 4). The acids projection toward the positive axis indicated their higher correlation with HRT20. This implied that the longer HRT promoted an overall increase in SCFAs concentration rather than changes in the individual acids profile. However, the CCA should be interpreted as an illustrative trend rather than a robust genus-conditions associated.

As it can be seen in Figure 4, despite the similarities in terms of bioconversion, the position of the reactors in the different quadrants may indicate a unique microbial composition and SCFAs profile. At phylum level, *Clostridium* and *Bifidobacterium* showed correlation with acetic and butyric production (Figure 4). *Bifidobacterium*, a lactic acid producer involved in carbohydrates degradation (Zhang et al., 2024), has been associated to butyric and caproic acids production as result of chain elongation (Zhang et al., 2023). The presence of lactic acid producers, like *Atopobium*, *Bifidobacterium*, or *Lactobacillus*, in the absence of lactic acid accumulation, suggested the conversion of this metabolite into acetyl-CoA followed by sequential chain elongation cycles, ultimately yielding butyric or caproic acids (Figures 1, 3) through reverse β-oxidation. *Acidaminococcus*, despite being considered a propionic and butyric acid producer, only showed slight correlation with even-chain acids (acetic, butyric and caproic acids). *Megasphaera* presented a preliminary correlation with HRT15 and butyric and acetic production (Figure 4). *Butyrivibrio*, a well-known butyric acid producer, was not associated with this acid in the CCA. The lack of association is attributed to the higher butyric acid concentration in HRT20, its lower relative abundance compared to other genera (e.g., *Clostridium*) and to the constrains imposed by the limited number of samples.

The SCFAs yield and the SCFAs distribution profile evidenced that the developed community depended on the selected HRT. The different microbial communities developed in the SBRs, dominated by *Clostridium*, *Atopobium*, and *Bulleidia*, corroborated that despite the fact that the HRT influenced the SCFAs concentration and distribution, no differences were observed in terms of bioconversion (> 55% in all cases). The s similar SCFAs profiles and bioconversions across reactors, despite microbial community shifts and VS_*in*_-driven differences in SCFAs concentration, indicate a strong functional robustness of the acidogenic community.

### 3.4 Participation of AK and BK in acetic and butyric acids production

In acidogenic microorganisms, the most widespread acetate-producing pathway is based on acetyl-CoA conversion into acetyl phosphate followed by its reversible de-phosphorylation, which is catalyzed by the AK (Kuit et al., 2012). Acetyl-CoA can also be subjected to sequential chain elongation cycles yielding butyric or caproic acids through reverse β-oxidation. Three more bacterial butyrate synthesis pathways have been identified, from glutarate, lysine or 4-aminobutyrate, but the four routes produce butyryl-CoA, which can be either phosphorylated and subsequently transformed to butyric acid by BK or directly converted into butyric acid by butyryl-CoA:acetate-CoA transferase (BUT) or other transferases (Esquivel-Elizondo et al., 2017; Gabris et al., 2015; Vital et al., 2014). Both AK and BK play a major role in the microbial energy metabolism, as their reaction also yields ATP from ADP, and thus they are often measured to investigate the acidogenesis efficiency under different conditions.

**FIGURE 5:**
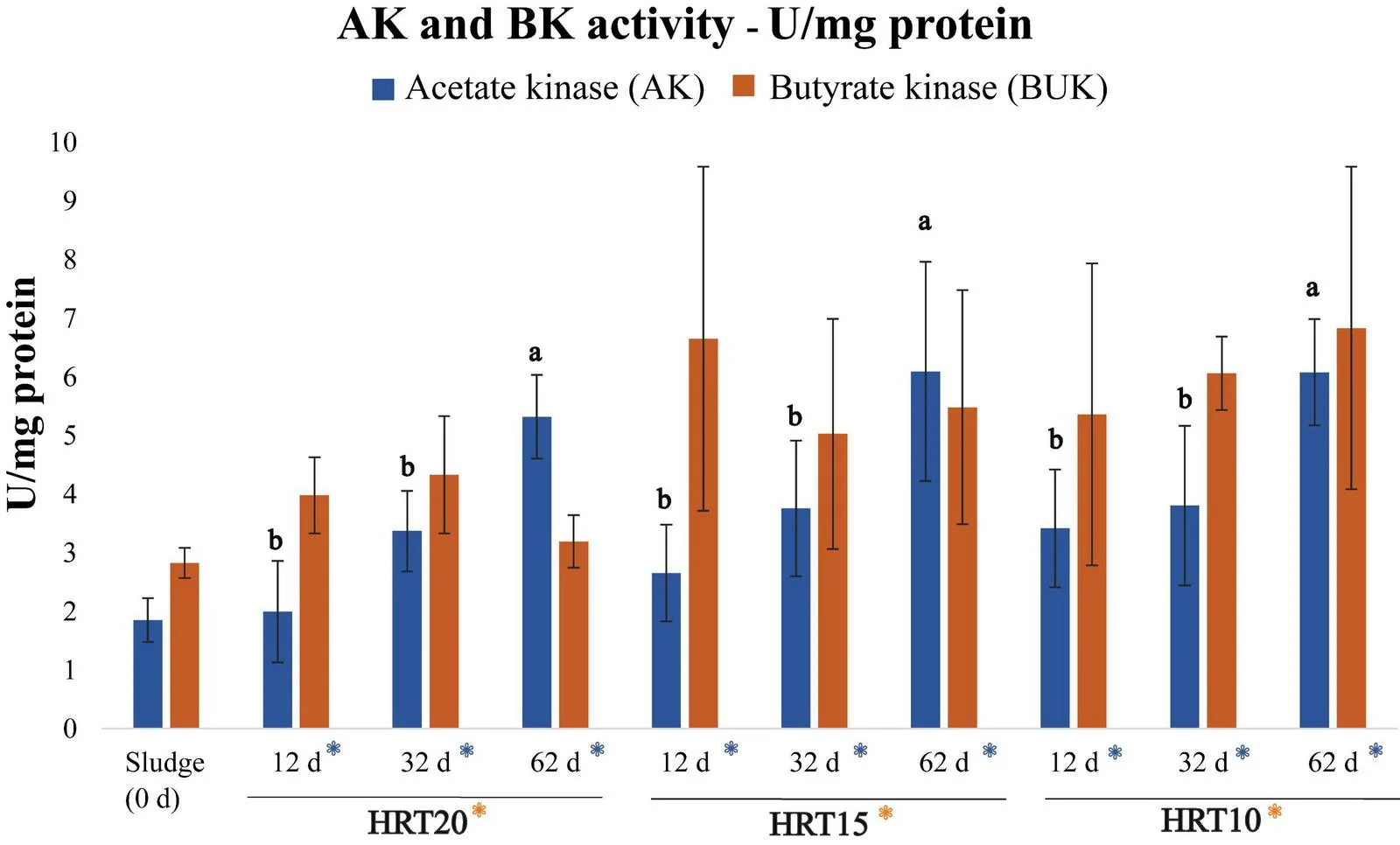
AK and BK activities in U/mg of total protein in cell free extracts throughout the three SBR reactors (0, 12, 32, and 62 d). Results represent the average of three independent, duplicated lysis experiments performed on different days. For AK, only the sampling day had a significant effect over the enzymatic activity (*p*-value < 0.05). The different letters indicate significant pairwise differences between day 62 and days 12 and 32 d (which did not differ). For BK, the shared letters (a, a, a) reflect the overall effect of the HRT in the two-way ANOVA (*p*-value < 0.05), but not differences were detected at the pairwise level (not shown in the figure). All the data met the assumptions for normality and homoscedasticity.

Herein, AK and BK were determined at three time points (12, 32 and 62 d) for all HRTs (10, 15 and 20) and for the initial sludge (Figure 5) by using cell free extracts after the lysis. Both enzymes were present in all samples, with specific activities ranging from 1.9 to 6.1 U/mg of total protein. AK activity showed a tendency to increase throughout the monitored time period, reaching a maximum of 5.3 ± 0.7, 6.1 ± 1.9 and 6.1 ± 0.9 U/mg of total protein after 62 d for HRT20, HRT15 and HRT10, respectively. A similar pattern was observed for the corresponding acetic acid levels during the stationary phase (22.6, 26.0, and 30.2% for HRT20, HRT15 and HRT10, respectively), indicating a well-functioning acidogenic step. The two-way ANOVA analysis evidenced that fermentation time significantly influenced AK activity, which could be attributed to the progressive adaptation of the microbial community. In contrast, the HRT had no significant effect, probably because the acetic concentration was similar in all reactors (Figure 5). AK activity may be correlated with the acetate-forming metabolism of *Clostridium* and *Bulleidia*, which are found in all three reactors and have been previously linked with AK production (Evseev et al., 2025; Kuit et al., 2012). Although AK activity has not been previously confirmed in *Atopobium*, its role in acetic acid production suggested that it may have contributed to the detected AK activity particularly for HRT15 and HRT10 (Figures 2, 4). AK activity in the sludge (0 d) was 1.85 ± 0.4 U/mg of total protein (Figure 5). This could be explained by its origin (from a WWTP), whereby the microbial communities while lower activity in biogas microbiomes reflects a system designed to consume acetate and convert it to methane. By opposite, in systems targeted at SCFAs accumulation, higher ACK activity microbiomes reflects a system designed to accumulate acetate.

BK activities were similar to those of AK, reaching an average of 3.2 ± 0.4, 5.4 ± 2 and 6.8 ± 2.7 U/mg of total protein for HRT20, HRT15 and HRT10, respectively (Figure 5), while the corresponding butyric acid levels were 53.5 ± 2.2, 48.4 ± 3.3 and 39.8 ± 2.5%. These results agree with the fact that BK is not the only enzyme catalyzing the last step in butyric acid synthesis, and thus BUT or other transferases are likely important contributors to the superior values of butyric acid found at all HRTs compared to the acetic acid ones (Vital et al., 2014).

As expected, BK activity during AF was higher than that of the sludge (2.8 ± 0.3 U/mg protein). HRT15 exhibited a higher BK activity than HRT20. This was confirmed by the ANOVA analysis that evidenced significant differences depending on the selected HRT, whereas the operational day had no significant relevance. The variability evidenced particularly for BK activity was attributed to the heterogeneity of the AF samples, as the centrifuged pellets contained both microbial cells and residual FW particles. Variations between aliquots may have influenced cell lysis, enzyme recovery and protein-normalized activity, however, the analysis design using three independent samples in duplicates per sampling point helped represent the heterogeneity and reduce the influence of an individual sample. Despite the variability significant, differences were still detected in the overall ANOVA. Although the main microbial difference in the former reactor was the presence of *Megasphaera*, no previous studies have reported BK activity associated to this genus. Instead, its butyrate production has been mainly linked to BUT. BK activity could be preliminary attributed to *Clostridium*, *Butyrivibrio*, and *Acidaminococcus*, which, as stated in section “3.3” are associated to butyrate production. Chen et al. (2017) and Gabris et al. (2015) reported BK activities below 0.03 U/mg of protein during AF and anaerobic digestion of organic wastes, respectively. Esquivel-Elizondo et al. (2017) found a low abundance of the genes encoding BK and BUT during AF despite being butyric acid the prevailing acid. Instead, encoding enzymes involved in lactate generation from pyruvate were found, suggesting a different mechanism for butyric acid production. This could be correlated with the presence of *Lactobacillus* and *Bifidobacterium* in the current study (Figure 3). Overall, this approach allows linking microbial composition with biochemical activity and microbial diversity, thereby providing a more comprehensive understanding of the pathways contributing to acetate and butyrate formation.

## 4 Conclusion

SBR configuration has been proved as a reliable alternative to CSTR for SCFAs production. In spite of the differences in HRT (20, 15 and 10 d), all SBRs achieved similar bioconversion efficiencies given the same high SRT. The highest SCFAs concentration (28.3 g/L) was reached at the longest HRT (20 d), however, higher productivities were obtained at HRT of 10 d (1.8 g SCFAs/Ld). The agricultural FW and selected operational conditions allowed a microbial metabolic versatility that mainly favored butyric and acetic acid accumulation. These predominant acids evidenced correlation with *Clostridium*, *Bifidobacterium*, *Atopobium*, and *Bulleidia*, aligned also with the enzymatic activity of AK and BK. Given the decantation step in SBR, this configuration enabled up to a one-third increase in the SCFAs yields in terms of g SCFAs/g TS in the supernatant, facilitating a subsequent downstream processing.

## Data Availability

The data that support the findings of this study are deposited in Zenodo (https://doi.org/10.5281/zenodo.18247081).

## Supplementary material

Supplementary Figure 1 Scheme of the SBR operated at different HRTs (20, 15 and 10 d). It represents the operating cycle steps (feeding, reaction, settle and withdraw). The withdraw volumes of each reactor are indicated.
