# Supplementary material for: Anaerobic sequencing batch reactor enhances short-chain fatty acids productivity while facilitating downstream processing
Source: Front Microbiol. 2026 Aug 19;17:1873229. doi: 10.3389/fmicb.2026.1873229 (PMC13533976; doi:10.3389/fmicb.2026.1873229)
Supplement: Supplementary file 1 [file Image_1.pdf]

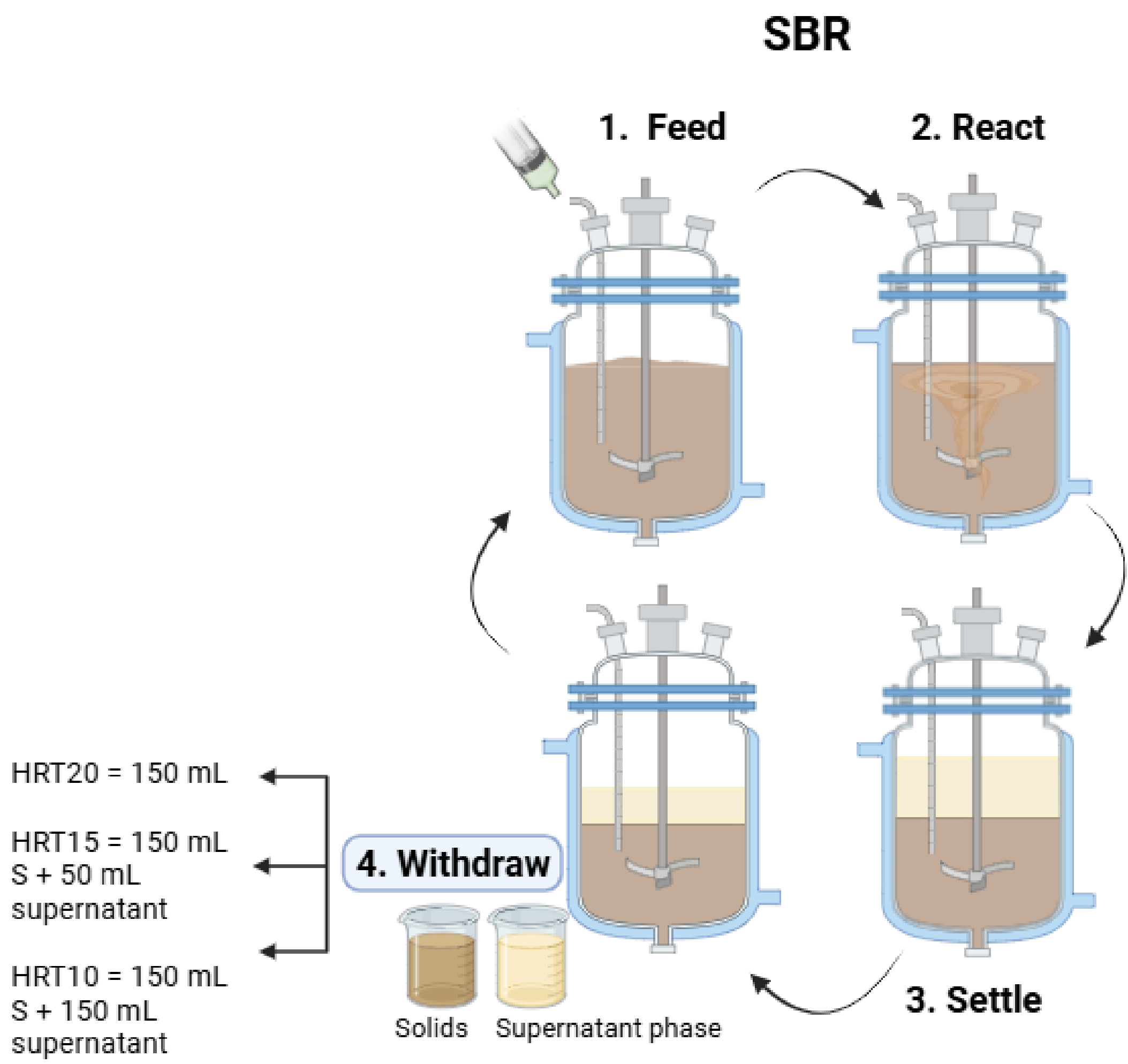
SBR
1. Feed
2. React
3. Settle
4. Withdraw
Solids
Supernatant phase
HRT20 = 150 mL
HRT15 = 150 mL S + 50 mL supernatant
HRT10 = 150 mL S + 150 mL supernatant
