# Supplementary material for: Anaerobic sequencing batch reactor enhances short-chain fatty acids productivity while facilitating downstream processing
Source: Front Microbiol. 2026 Aug 19;17:1873229. doi: 10.3389/fmicb.2026.1873229 (PMC13533976; doi:10.3389/fmicb.2026.1873229)
Supplement: Supplementary file 2 [file Table_1.docx]

**Table S1.** Archaea and bacterial relative abundance at phylum and genus level

**a)**

| **Phylum** | | | | | | | |  |  |  |  |  |
| --- | --- | --- | --- | --- | --- | --- | --- | --- | --- | --- | --- | --- |
|  | **Inoculum** | **HRT20** | **HRT15** | | | **HRT10** | | |  |  |  |  |
| Unassigned;Other | 1.30 | 0.11 | | 1.03 | 3.85 | |  |  |  |  |  |  |
| k__Archaea;p__Euryarchaeota | 1.88 | 4.32 | | 3.23 | 2.33 | |  |  |  |  |  |  |
| k__Bacteria;p__Actinobacteria | 16.51 | 13.79 | | 22.68 | 29.46 | |  |  |  |  |  |  |
| k__Bacteria;p__Bacteroidetes | 16.74 | 0.02 | | 0.17 | 1.41 | |  |  |  |  |  |  |
| k__Bacteria;p__Caldiserica | 1.33 | 0.00 | | 0.00 | 0.00 | |  |  |  |  |  |  |
| k__Bacteria;p__Chloroflexi | 10.44 | 0.03 | | 0.01 | 0.00 | |  |  |  |  |  |  |
| k__Bacteria;p__Firmicutes | 16.81 | 81.45 | | 72.64 | 62.80 | |  |  |  |  |  |  |
| k__Bacteria;p__NKB19 | 0.80 | 0.00 | | 0.00 | 0.00 | |  |  |  |  |  |  |
| k__Bacteria;p__OD1 | 2.32 | 0.00 | | 0.00 | 0.00 | |  |  |  |  |  |  |
| k__Bacteria;p__OP9 | 0.68 | 0.00 | | 0.00 | 0.00 | |  |  |  |  |  |  |
| k__Bacteria;p__Planctomycetes | 3.33 | 0.02 | | 0.00 | 0.00 | |  |  |  |  |  |  |
| k__Bacteria;p__Proteobacteria | 12.82 | 0.18 | | 0.22 | 0.13 | |  |  |  |  |  |  |
| k__Bacteria;p__Spirochaetes | 0.65 | 0.00 | | 0.00 | 0.00 | |  |  |  |  |  |  |
| k__Bacteria;p__Synergistetes | 3.77 | 0.02 | | 0.00 | 0.00 | |  |  |  |  |  |  |
| k__Bacteria;p__TM7 | 0.72 | 0.00 | | 0.00 | 0.00 | |  |  |  |  |  |  |
| k__Bacteria;p__Verrucomicrobia | 1.30 | 0.00 | | 0.00 | 0.00 | |  |  |  |  |  |  |
| k__Bacteria;p__WS6 | 2.22 | 0.00 | | 0.00 | 0.00 | |  |  |  |  |  |  |
| k__Bacteria;p__WWE1 | 3.74 | 0.00 | | 0.00 | 0.00 | |  |  |  |  |  |  |
|  | | | | | | |  | | |  |  |  |
| **b)** | | | | | | |  | | |  |  |  |
| **Genus** | | | | | | | | | | | | |
|  | | | | | | | **Inoculum** | | | **HRT20** | **HRT15** | **HRT10** |
| Unassigned;Other;Other;Other;Other;Other | | | | | | | 29.00 | | | 4.60 | 2.91 | 6.48 |
| k__Archaea;p__Euryarchaeota;c__Methanobacteria;o__Methanobacteriales;f__Methanobacteriaceae;g__Methanobrevibacter | | | | | | | 0.02 | | | 2.21 | 1.87 | 1.32 |
| k__Archaea;p__Euryarchaeota;c__Methanobacteria;o__Methanobacteriales;f__Methanobacteriaceae;g__Methanosphaera | | | | | | | 0.00 | | | 2.10 | 1.36 | 1.01 |
| k__Archaea;p__Euryarchaeota;c__Methanomicrobia;o__Methanosarcinales;f__Methanosaetaceae;g__Methanosaeta | | | | | | | 1.61 | | | 0.00 | 0.00 | 0.00 |
| k__Bacteria;p__Actinobacteria;c__Actinobacteria;o__Actinomycetales;f__;g__ | | | | | | | 2.02 | | | 0.00 | 0.00 | 0.00 |
| k__Bacteria;p__Actinobacteria;c__Actinobacteria;o__Actinomycetales;f__Dietziaceae;g__Dietzia | | | | | | | 3.31 | | | 0.00 | 0.00 | 0.00 |
| k__Bacteria;p__Actinobacteria;c__Actinobacteria;o__Actinomycetales;f__Intrasporangiaceae;g__ | | | | | | | 4.00 | | | 0.00 | 0.00 | 0.00 |
| k__Bacteria;p__Actinobacteria;c__Actinobacteria;o__Actinomycetales;f__Microbacteriaceae;g__Leucobacter | | | | | | | 1.09 | | | 0.03 | 0.00 | 0.00 |
| k__Bacteria;p__Actinobacteria;c__Actinobacteria;o__Actinomycetales;f__Mycobacteriaceae;g__Mycobacterium | | | | | | | 2.19 | | | 0.00 | 0.00 | 0.00 |
| k__Bacteria;p__Actinobacteria;c__Actinobacteria;o__Bifidobacteriales;f__Bifidobacteriaceae;g__ | | | | | | | 0.00 | | | 1.03 | 1.94 | 2.91 |
| k__Bacteria;p__Actinobacteria;c__Actinobacteria;o__Bifidobacteriales;f__Bifidobacteriaceae;g__Bifidobacterium | | | | | | | 0.06 | | | 2.86 | 1.83 | 1.45 |
| k__Bacteria;p__Actinobacteria;c__Coriobacteriia;o__Coriobacteriales;f__Coriobacteriaceae;g__ | | | | | | | 0.03 | | | 2.39 | 1.96 | 3.10 |
| k__Bacteria;p__Actinobacteria;c__Coriobacteriia;o__Coriobacteriales;f__Coriobacteriaceae;g__Atopobium | | | | | | | 0.00 | | | 6.66 | 16.10 | 21.78 |
| k__Bacteria;p__Bacteroidetes;c__Bacteroidia;o__Bacteroidales;f__;g__ | | | | | | | 12.31 | | | 0.00 | 0.00 | 0.00 |
| k__Bacteria;p__Bacteroidetes;c__Bacteroidia;o__Bacteroidales;f__Prevotellaceae;g__Prevotella | | | | | | | 0.00 | | | 0.00 | 0.00 | 0.00 |
| k__Bacteria;p__Bacteroidetes;c__Bacteroidia;o__Bacteroidales;f__SB-1;g__ | | | | | | | 3.01 | | | 0.00 | 0.00 | 0.00 |
| k__Bacteria;p__Caldiserica;c__OP5;o__;f__;g__ | | | | | | | 1.12 | | | 0.00 | 0.00 | 0.00 |
| k__Bacteria;p__Chloroflexi;c__Anaerolineae;o__Anaerolineales;f__Anaerolinaceae;g__ | | | | | | | 3.85 | | | 0.00 | 0.00 | 0.00 |
| k__Bacteria;p__Chloroflexi;c__Anaerolineae;o__Anaerolineales;f__Anaerolinaceae;g__T78 | | | | | | | 3.36 | | | 0.00 | 0.00 | 0.00 |
| k__Bacteria;p__Chloroflexi;c__Anaerolineae;o__Caldilineales;f__Caldilineaceae;g__Caldilinea | | | | | | | 1.35 | | | 0.00 | 0.00 | 0.00 |
| k__Bacteria;p__Firmicutes;c__Bacilli;o__Lactobacillales;f__;g__ | | | | | | | 0.00 | | | 0.00 | 4.03 | 0.00 |
| k__Bacteria;p__Firmicutes;c__Clostridia;o__Clostridiales;Other;Other | | | | | | | 2.21 | | | 0.00 | 0.00 | 0.00 |
| k__Bacteria;p__Firmicutes;c__Clostridia;o__Clostridiales;f__;g__ | | | | | | | 2.82 | | | 59.56 | 35.52 | 29.11 |
| k__Bacteria;p__Firmicutes;c__Clostridia;o__Clostridiales;f__Christensenellaceae;g__ | | | | | | | 1.01 | | | 0.00 | 0.00 | 0.00 |
| k__Bacteria;p__Firmicutes;c__Clostridia;o__Clostridiales;f__Clostridiaceae;g__ | | | | | | | 1.75 | | | 0.00 | 0.00 | 0.00 |
| k__Bacteria;p__Firmicutes;c__Clostridia;o__Clostridiales;f__Eubacteriaceae;g__Pseudoramibacter_Eubacterium | | | | | | | 0.02 | | | 0.00 | 0.00 | 2.87 |
| k__Bacteria;p__Firmicutes;c__Clostridia;o__Clostridiales;f__Lachnospiraceae;g__Butyrivibrio | | | | | | | 0.00 | | | 3.79 | 5.36 | 4.61 |
| k__Bacteria;p__Firmicutes;c__Clostridia;o__Clostridiales;f__Ruminococcaceae;g__ | | | | | | | 1.06 | | | 0.00 | 0.00 | 0.00 |
| k__Bacteria;p__Firmicutes;c__Clostridia;o__Clostridiales;f__Veillonellaceae;g__Acidaminococcus | | | | | | | 0.00 | | | 1.94 | 3.34 | 1.90 |
| k__Bacteria;p__Firmicutes;c__Clostridia;o__Clostridiales;f__Veillonellaceae;g__Megasphaera | | | | | | | 0.00 | | | 0.00 | 12.24 | 1.23 |
| k__Bacteria;p__Firmicutes;c__Clostridia;o__Clostridiales;f__[Mogibacteriaceae];g__ | | | | | | | 1.53 | | | 0.00 | 0.00 | 0.00 |
| k__Bacteria;p__Firmicutes;c__Clostridia;o__Clostridiales;f__[Tissierellaceae];g__Sedimentibacter | | | | | | | 1.97 | | | 0.00 | 0.00 | 0.00 |
| k__Bacteria;p__Firmicutes;c__Erysipelotrichi;o__Erysipelotrichales;f__Erysipelotrichaceae;g__Bulleidia | | | | | | | 0.00 | | | 9.09 | 7.37 | 17.36 |
| k__Bacteria;p__OD1;c__ABY1;o__;f__;g__ | | | | | | | 1.92 | | | 0.00 | 0.00 | 0.00 |
| k__Bacteria;p__Planctomycetes;c__Planctomycetia;o__Pirellulales;f__Pirellulaceae;g__ | | | | | | | 1.08 | | | 0.00 | 0.00 | 0.00 |
| k__Bacteria;p__Planctomycetes;c__Planctomycetia;o__Planctomycetales;f__Planctomycetaceae;g__Planctomyces | | | | | | | 1.12 | | | 0.00 | 0.00 | 0.00 |
| k__Bacteria;p__Proteobacteria;c__Alphaproteobacteria;o__Rhizobiales;f__;g__ | | | | | | | 1.06 | | | 0.00 | 0.00 | 0.00 |
| k__Bacteria;p__Proteobacteria;c__Alphaproteobacteria;o__Rhodobacterales;f__Rhodobacteraceae;g__ | | | | | | | 1.97 | | | 0.00 | 0.00 | 0.00 |
| k__Bacteria;p__Proteobacteria;c__Betaproteobacteria;o__Burkholderiales;f__Comamonadaceae;g__ | | | | | | | 1.78 | | | 0.00 | 0.00 | 0.00 |
| k__Bacteria;p__Synergistetes;c__Synergistia;o__Synergistales;f__Dethiosulfovibrionaceae;g__HA73 | | | | | | | 1.54 | | | 0.00 | 0.00 | 0.00 |
| k__Bacteria;p__Synergistetes;c__Synergistia;o__Synergistales;f__Synergistaceae;g__vadinCA02 | | | | | | | 1.26 | | | 0.00 | 0.00 | 0.00 |
| k__Bacteria;p__WS6;c__SC72;o__A-2AF;f__;g__ | | | | | | | 1.08 | | | 0.00 | 0.00 | 0.00 |
| k__Bacteria;p__WS6;c__SC72;o__WCHB1-15;f__;g__ | | | | | | | 1.14 | | | 0.00 | 0.00 | 0.00 |
| k__Bacteria;p__WWE1;c__[Cloacamonae];o__[Cloacamonales];f__[Cloacamonaceae];g__Candidatus Cloacamonas | | | | | | | 1.46 | | | 0.00 | 0.00 | 0.00 |
| k__Bacteria;p__WWE1;c__[Cloacamonae];o__[Cloacamonales];f__[Cloacamonaceae];g__W22 | | | | | | | 1.94 | | | 0.00 | 0.00 | 0.00 |
